# ^64^Cu-ATSM/^64^Cu-Cl_2_ and their relationship to hypoxia in glioblastoma: a preclinical study

**DOI:** 10.1186/s13550-019-0586-6

**Published:** 2019-12-19

**Authors:** Elodie A. Pérès, Jérôme Toutain, Louis-Paul Paty, Didier Divoux, Méziane Ibazizène, Stéphane Guillouet, Louisa Barré, Aurélien Vidal, Michel Cherel, Mickaël Bourgeois, Myriam Bernaudin, Samuel Valable

**Affiliations:** 1 Normandie Univ, UNICAEN, CEA, CNRS, ISTCT/CERVOxy group, GIP Cyceron, Caen, France; 2 Nantes-Angers Cancer Research Center CRCINA, University of Nantes, INSERM UMR1232, CNRS-ERL6001, Nantes, France; 3 Normandie Univ, UNICAEN, CEA, CNRS, ISTCT/LDM-TEP group, GIP Cyceron, Caen, France; 4 GIP ARRONAX, Nantes, France; 5 Nuclear Medicine Department, ICO-René Gauducheau Cancer Center, Saint-Herblain, France; 6 Nuclear Medicine Department, University Hospital, Nantes, France

**Keywords:** Hypoxia, Brain tumors, Cu-ATSM, PET radiotracers, Copper transporters

## Abstract

**Background:**

Diacetyl-bis(N4-methylthiosemicarbazone), labeled with 64Cu (^64^Cu-ATSM) has been suggested as a promising tracer for imaging hypoxia. However, various controversial studies highlighted potential pitfalls that may disable its use as a selective hypoxic marker. They also highlighted that the results may be tumor location dependent. Here, we first analyzed uptake of Cu-ATSM and its less lipophilic counterpart Cu-Cl_2_ in the tumor over time in an orthotopic glioblastoma model. An in vitro study was also conducted to investigate the hypoxia-dependent copper uptake in tumor cells. We then further performed a comprehensive ex vivo study to compare ^64^Cu uptake to hypoxic markers, specific cellular reactions, and also transporter expression.

**Methods:**

μPET was performed 14 days (^18^F-FMISO), 15 days (^64^Cu-ATSM and ^64^Cu-Cl2), and 16 days (^64^Cu-ATSM and ^64^Cu-Cl_2_) after C6 cell inoculation. Thereafter, the brains were withdrawn for further autoradiography and immunohistochemistry. C6 cells were also grown in hypoxic workstation to analyze cellular uptake of Cu complexes in different oxygen levels.

**Results:**

In vivo results showed that Cu-ASTM and Cu-Cl2 accumulated in hypoxic areas of the tumors. Cu-ATSM also stained, to a lesser extent, non-hypoxic regions, such as regions of astrogliosis, with high expression of copper transporters and in particular DMT-1 and CTR1, and also characterized by the expression of elevated astrogliosis. In vitro results show that 64Cu-ATSM showed an increase in the uptake only in severe hypoxia at 0.5 and 0.2% of oxygen while for ^64^Cu-Cl2, the cell retention was significantly increased at 5% and 1% of oxygen with no significant rise at lower oxygen percentages.

**Conclusion:**

In the present study, we show that Cu-complexes undoubtedly accumulate in hypoxic areas of the tumors. This uptake may be the reflection of a direct dependency to a redox metabolism and also a reflection of hypoxic-induced overexpression of transporters. We also show that Cu-ATSM also stained non-hypoxic regions such as astrogliosis.

## Background

Hypoxia, resulting from an inadequacy between oxygen supply and demand, is noticeably pronounced in various solid tumors such as glioblastoma (GB). While in the normal brain oxygen pressure ranges between 30 and 60 mmHg, it is suspected to be less than 10 mmHg in hypoxic brain tumors and can be less than 1 mmHg in some tumor areas [1, 2].

At the cellular level, hypoxia triggers numerous responses, in part mediated by the activation of the family of hypoxia-inducible factors (HIFs). HIFs are transcription factors that, in turn, induce the expression of various genes involved in several processes known to participate in tumor growth such as angiogenesis, regulation of intracellular pH, regulation of oxidative metabolism, glycolytic metabolism, invasion, and apoptosis. As a whole, hypoxia and the activation of the HIFs transcription factor is a factor of poor prognosis, in the context of solid tumors and particularly in glioblastoma [3, 4].

Hypoxia also induces resistance to anti-cancer treatments. Original experiments in the early 50s already demonstrated the importance of the oxygen effect in X-rays’ efficacy with the generation of reactive oxygen species. Thereby, hypoxia is involved in tumor radioresistance [5]. In addition, the efficacy of various chemotherapies is also linked to the presence of oxygen [6, 7].

At the tissue level, it is now demonstrated that hypoxia is not a binary effect and that intratumor gradients exist. The concept of diffusion limited hypoxia also referred to as chronic hypoxia (i.e., the ability of oxygen to diffuse from the nearest capillary, which represents a distance of ~ 70 μm) was described 70 years ago. More recently, the concept of perfusion limited hypoxia (also referred to as acute and cycling, dynamic hypoxia) resulting from blood obstruction, altered blood flow, poorly oxygenated blood, and increased transit time has been proposed [8]. The observation that hypoxia is heterogeneous over the tumor mass and also in time opens the avenue for more adapted treatments requiring more specific and/or sensitive imaging of hypoxia.

Various methods have been proposed to map hypoxia (see [9] for review) and various radiopharmaceuticals that could be used for positron emission tomography (PET) have been proposed. As examples, nitro-imidazole compounds have been extensively studied and 3-^18^F-fluoro-1-(2-nitro-1-imidazolyl)-2-propanol (^18^F-FMISO) has appeared as one of the most efficient tracers to non-invasively map hypoxia in brain tumors. Nitro-imidazole compounds enter in all cells where it suffers reductions. It is not a stable compounds in this state and then is either reoxidized if oxygen is present or binds to macromolecule if not oxygen is present in the cell. However, ^18^F-FMISO suffers from some drawbacks. It is sensitive to severe hypoxia (less than 10 mmHg) and fails to reveal regions with moderate to mild hypoxia [10]. FMISO may be sensitive to low potential redox. A low tumor-to-background contrast resulting from a low clearance in normoxic tissue and a low lipophilia is also an additional drawback. Moreover, the short half-life of ^18^F (109 min) also raises the problem of large-scale spread of this hypoxia imaging marker. In contrast, while the relationship between ^18^F-FMISO uptake and ptO_2_ is not linear, it is a robust and reproducible hypoxic marker thanks to its nitro-imidazole moiety.

More recently, diacetyl-bis(N4-methylthiosemicarbazone), labeled with various positron-emitting isotopes of copper, such as ^64^Cu-diacetyl-bis(N4-methylthiosemicarbazone) (^64^Cu-ATSM), has been suggested as a promising tracer for imaging hypoxia due to its high membrane permeability and low redox potential [11, 12]. ATSM confers lipophilic properties to the molecule in comparison to ^64^Cu-Cl_2_. In the context of brain tumors, a good correlation was observed between ^64^Cu-ATSM and ^18^F-FMISO PET imaging [13], and also between high ^64^Cu-ATSM uptake and low pO2 [14]. Significant correlations between ^62^Cu-ATSM and HIF staining were also observed [15] in clinical situation, and this tracer has been recently proposed as an independent prognostic biomarker of survival in high-grade glioma [16]. However, the selectivity of ^64^Cu-ATSM to hypoxia has been challenged. As an example, in a study comparing four tracers of hypoxia in a single xenograft model chosen for its regional heterogeneity, ^64^Cu-ATSM failed to correlate to pimonidazole and carbonic anhydrase IX (CAIX) staining while the other three tracers did [17]. Likewise, studies on the microscopic intratumor distribution of ^64^Cu-ATSM showed no correlation between this radiotracer and hypoxia marker (pimonidazole) or perfusion marker (Hoechst-33342) [18]. Moreover, in the presence of hypercapnic gasses known to reduce intratumor hypoxia, Yuan and Colleagues failed to show significant changes in the uptake of Cu-ATSM [19]. It is worth mentioning that all experiments conducted on brain tumor model have been performed ectopically (in the flank) so far, and it is therefore necessary to study whether Cu-ATSM uptake is specific for hypoxia in an orthotopic model. Consequently, debates remain on the selectivity of ^64^Cu-ATSM to hypoxia, and several preclinical studies have focused on better understanding uptake and retention under hypoxic conditions.

Fujibayashi and his collaborators first proposed that the reduction of Cu(II) in Cu(I) would only occur in hypoxic cells because of the abnormally reduced state of their mitochondria [12]. Furthermore, it was also demonstrated that NADH and NADPH [12, 20] and also over-reduced states caused by mitochondrial dysfunction even in normoxia [21] were involved in the production of Cu(I). Cu(I) would dissociate slowly from the ATSM ligand in cells with low oxygen concentration, becoming irreversibly trapped. Intriguingly, Dearling and colleagues observed that retention of Cu-ATSM was partially reversible in hypoxic cells [22]. The selectivity to hypoxia would therefore be a competition between dissociation and re-oxidation of the reduced copper complexes. Holland and colleagues also proposed a revised mechanism that may influence the degree of Cu-ASTM uptake based on protonation leading to intracellular release of the copper ion and thereby forms mono-or di-protonated complexes which governs the in vivo behavior [23].

Burgman and colleagues demonstrated the efflux and decrease in ^64^Cu-ATSM in anoxic and hypoxic prostate tumor cells, in contradiction with models that suggest irreversible trapping under low-oxygen concentration conditions [24]. The authors proposed that after dissociation of Cu(I) and ATSM, Cu(I) would not be trapped but absorbed in the intracellular pool of copper and be subjected to cellular metabolism of copper. The level of expression and regulation of its transporters could vary with cell types, which may explain variations in copper retention. Thus, membrane transporters such as multidrug resistance protein 1 (MDR1) or other specific copper transporters in particular copper transporter 1 (CTR1) and divalent metal transporter 1 (DMT1) could also influence the retention of copper [25].

All together, these data suggest that further experiments are required to further assess the specificity of ^64^Cu-ATSM as an imaging marker of hypoxia, in particular in the context of brain tumors. Here, the selectivity of ^64^Cu-ATSM with respect to hypoxia was studied, at the preclinical level, in a glioblastoma (GB) model. By combining different experimental approaches, the specificity of ^64^Cu-ATSM for hypoxia was evaluated in vivo by PET imaging and compared to ^18^F-FMISO (the reference radiotracer for hypoxia) and ^64^Cu-Cl_2_ (used as a control of copper). In addition, cell retention of ^64^Cu-ATSM and ^64^Cu Cl_2_ were determined in vitro in various oxygenation conditions. Imaging results were also supported by ex vivo autoradiography studies of brain sections of GB-bearing rats and immunolabeling for different hypoxia-induced proteins and copper transporters.

## Methods

### In vivo studies

#### Ethics statement

The animal investigations were performed under the current European directive (2010/63/EU) in authorized laboratories (B14118001) and with the permission of the regional committee on animal ethics (CENOMEXA, APAFIS#1247). Data are reported according to ARRIVE guidelines. The global protocol is detailed l in Additional file 1: Figure S1.

#### Tumor models

The C6 cell line was purchased from ATCC (American Type Culture Collection). Tumor cells were cultured in DMEM 4.5 g/l glucose (Sigma-Aldrich, France) supplemented with 10% fetal calf serum (FCS) (InVitrogen, France), 2 mM glutamine (Gln) (Sigma-Aldrich, France), and 100 U/mL penicillin/streptomycin (PS) (InVitrogen, France). Male Wistar rats (6–8 weeks old, *n* = 6 for PET 18F-FMISO/^64^Cu-ATSM and *n* = 11 for Cu-Cl_2_) were purchased from Janvier Labs (Le Genest-Saint-Isle, France). Isoflurane anesthesia was used for the rats (5% for induction and 2% for maintenance in 70% N_2_O/30% O_2_). Rectal temperature was monitored and maintained around 37.0 °C throughout the experiments as already described [26]. Rats were placed on a stereotactic head holder and a sagittal scalp incision was performed. A 1-mm diameter burr hole was drilled in the calvarium, 3 mm lateral to the bregma. C6 (1.10^5^), in 3-μl PBS-glutamine 2 mM were injected over 6 min via a fine needle (30 G) connected to a Hamilton syringe. The injection site was the right caudate-putamen, at a depth of 6 mm beneath the calvarium. The needle was removed slowly 5 min after the end of the injection, and the burr hole was sealed with dental cement.

#### Magnetic resonance imaging (MRI)

MRI was performed on day 2 using a 7-T horizontal magnet (Bruker, Ettlingen, Germany; CYCERON biomedical imaging platform). A cross coil configuration was used (volume/surface coil, Bruker, Ettlingen, Germany).

After scout imaging, an anatomical exploration of the brain was performed using a T2w sequence (RARE, acceleration factor of 8; TR/TE_eff_ = 5000/62.5 ms; Number of EXperiments (NEX) = 1; 20 contiguous slices; resolution = 0.15 × 0.15 × 0.75 mm; acquisition time = 2 min). TR and TE are respectively repetition time and echo time.

#### Positron emission tomography (PET)

On day 1, ^18^F-FMISO (produced by the LDM-TEP group, ISTCT) was intravenously injected in physiological saline (≈ 66 MBq.kg^−1^). At *t* = 1.5 h, the rats were transferred into the PET camera (Inveon®, Siemens, Erlangen, Germany; CYCERON biomedical imaging platform). An X-ray scan was first performed to obtain attenuation maps just prior to an emission scan of 20 min initiated at *t* = 2 h.

On day 0, ^64^Cu-ATSM (287 μCi/nmol) or ^64^Cu-Cl_2_ (276 μCi/nmol) (produced by ARRONAX, Nantes, France) were intravenously injected (≈ 90 MBq.kg^−1^), and PET imaging were performed at 3 h and 24 h (day+1) after the attenuation acquisition. All images were reconstructed by the iterative OSEM-2D algorithm. For some animals, a dynamic acquisition lasting 2 h and 20 min was performed. In parallel, blood samples were withdrawn for arterial input function determination over a period of 2 h.

Following the late acquisition (i.e., 24 h post-injection), rats were injected with a pimonidazole solution (Hypoxyprobe®-1, Hypoxyprobe Incorporation, USA) of 80 mg/kg i.p., 120 min before the animals were euthanized under deep anesthesia just after the completion of PET imaging examinations. Following the last PET session, the rat brains were withdrawn and immediately snap-frozen for subsequent autoradiography and immunohistochemistry.

### Ex vivo studies

#### Autoradiography

Series of coronal sections (20 μm) were cut on a cryostat (Leica, Germany). Digital autoradiography was prepared by placing tissue section in a film cassette against a phosphor imaging plate for an overnight exposure. Phosphor imaging plates were then read at a pixel resolution of 50 μm with a Cyclone analyzer (PerkinElmer). Adjacent slices were used for immunohistochemistry studies.

#### Immunohistochemistry

First, slices were post-fixated in cold acetone for 10 min, then the nonspecific binding sites were blocked by bovine serum albumin (BSA) 3%/Tween 0.1%/Triton 0.5% in PBS solution for 90 min at room temperature. The slices were incubated overnight with primary antibodies at 4 °C in BSA 1%/Tween 0.1%/Triton 0.5% in PBS solution (Additional file 2: Table S1), and the staining was revealed by fluorochrome-conjugated secondary antibodies (Additional file 2: Table S1). Nuclei were counterstained with Hoechst 33342 (Sigma-Aldrich, 10 μg/ml). Tissue sections were examined at × 10 magnification with a Leica DM6000, and whole brain slices were reconstructed using mosaic acquisitions (MetaMorph software).

#### Quantification of immunohistochemistry

After co-registration of immunohistochemistry and autoradiography images for all rats (*n* = 9), a visual inspection was used for each region named (R1, R2, and R3 as presented on Fig. 4a). For each staining, two parameters were quantified: the number of positive samples determined when staining was visually detected and the staining intensity by assigning a score equivalent to 1 or 2 for low and high expression, respectively. Then, a global score, including all rats, was measured for each staining by multiplying the number of positive samples and total score of intensity staining.

### Data analysis

#### Image processing and analysis

Image analysis was performed with in-house macros based on the ImageJ software (http://rsb.info.nih.gov/ij/, 1997-2012). PET analyses were performed with PMOD 3.1 software [27].

##### PET

To quantify ^18^F-FMISO, ^64^Cu-ATSM, and ^64^Cu-Cl_2_ uptake, the measured concentration of tissue activity (counts, kBq/mL) were divided by the injected activity in kBq/g of body weight to give a standardized uptake value (SUV, g/mL).

##### Tumor delineation

The regions of interest corresponding to the tumor and the contralateral mirror tissue were manually delineated on all contiguous T2w slices. The ROI corresponding to the tumor or to the contralateral tissue was used thereafter for measurements of all other parameters. These ROIs defined on MRI were used for comparison between radiotracers.

##### MRI/PET co-registration

All MRI experiments were performed such that all MRI maps were anatomically colocalized. A first automatic registration (PMOD 3.1) was performed between T2w MRI (reference) and the X-ray scan (input) using a rigid registration algorithm based on mutual information. The transformation matrix so obtained was then applied to all PET emission scans. Registration was visually assessed and, when necessary, manually refined.

##### Image segmentation

A ROI in the contralateral tissue was manually delineated to obtain a mean ± SD value. Then, a mean ± 3.3 SD threshold was used for segmentation to obtain a hypoxic volume for both 18F-FMISO and ^64^Cu-ATSM. These segmented ROIs were used for spatial analyses between both tracers.

### In vitro studies

In vitro studies were carried out on C6 glioblastoma cells for which the culture conditions have been previously described. Once at confluence, the cells were seeded at a density of 200,000 cells/mL in T25 flask 48 h prior to exposure to radiotracers.

#### In vitro uptake in hypoxic conditions

To study the uptake of ^64^Cu-ATSM and ^64^Cu-Cl_2_ by hypoxic cells, various oxygenation protocols were studied which were intended to modify various parameters: the degree of oxygenation (21%, 5%, 1%, 0.5%, and 0.2% O_2_), hypoxia preconditioning time (3 h and 24 h), and radiopharmaceutical exposure time (1 h and 4 h).

During exposures to ^64^Cu-ATSM or ^64^Cu-Cl_2_ that lasted 1 h or 4 h, the cells were cultured in the same oxygenation condition used before treatment. A dose of 0.148 MBq (4 μCi) was provided in a volume of 2 mL of medium for each culture dish, both for of ^64^Cu-ATSM (457 μCi/nmol) and ^64^Cu-Cl_2_ (453 μCi/nmol). For each experimental condition, three independent cell cultures were analyzed.

^64^Cu retention was then assessed in various samples withdrawn from dishes: cell supernatant (2 mL), washing buffer with PBS (2 mL), and cell lysate (prepared from 200 μL of RIPA buffer).

#### Counting radioactivity

Radioactivity was counted in 1 mL of supernatant derived from cell cultures, in 100 μL of cell lysate, and in 1 mL of washing and on the pipetting cones, by a gamma counter (Wizard). All calculations were expressed as the percentage of radionuclide incorporated in the cells (relative to the dose delivered). To avoid effects probably due to different cell number in culture flasks, the cellular uptake was then normalized by the biomass and express as % cell retention/mg protein. Cells were lysed by mechanical dissociation (scraping) on ice in RIPA buffer. The supernatants were then removed to recover total cell proteins using a spectrophotometric colorimetric assay (OD measured at 562 nm; Pierce BCA Protein Assay Kit from Thermo-Scientific, France).

#### RT-qPCR analysis

RNA was withdrawn using Nucleospin® RNA II Kit (Macherey-Nagel, France) according to the manufacturer’s protocol. One microgram of total RNA from each sample was reverse-transcribed using the Promega RT system (Promega, France) (RT at 42 °C for 1 h). Forward (F) and reverse (R) primers were designed for each gene using Beacon Designer software (Bio-Rad, France) as detailed in Additional file 2: Table S2. Assays were run in duplicate on the iCycler iQ™ realtime PCR detection system (Bio-Rad, France). The amplification profile was as follows: Hot Goldstar enzyme activation, 95 °C for 3 min; PCR 50 cycles at 95 °C, 15 s, and 60 °C, 1 min. The PCR was done according to the manufacturer’s protocol using the PCR™ Core Kit Sybr™ Green I (Eurogentec, France). The results were analyzed using a comparative method between the fractional cycle number to reach a fixed threshold and the fractional cycle number of β-actin gene and expressed using the 2^−ΔCt^ formula.

#### Western blot analysis

The remaining cell lysate used for in vitro uptake of radioactivity were then processed for western blot analyses. Cells exposed to decreasing percentage of oxygen were lysed with RIPA buffer (Sigma-Aldrich, France) supplemented with 1 μg/mL protease inhibitors (Sigma-Aldrich, France) and 1 μg/mL phosphatase inhibitors (Sigma-Aldrich, France). Proteins (40 μg) were separated by SDS-PAGE and transferred to polyvinylidene difluoride membranes (GE Healthcare Bio-Sciences, Sweden). The protein expression of CAIX, CTR1, and DMT1 were evaluated by specific primary antibodies (Additional file 2: Table S1), and β-Actin expression was used to check the equal loading in proteins on the gel. Blots were exposed to peroxidase-linked secondary antibodies (Additional file 2: Table S1), and immunoreactive bands were visualized by enhanced chemiluminescence reagents (Thermo-Scientific, France). The band intensity was processed with ImageJ and was normalized to their corresponding actin signal. Western blot photographs are representative of three independent experiments.

### Statistical analyses

All data are presented as mean ± SD, unless otherwise stated. Statistical analyses were performed with JMP® program (SAS Institute Inc., USA). The different tests used are detailed in each figure legend.

## Results

### In vivo uptake of Cu-ATSM and Cu-Cl_2_ in a GB model

In order to investigate the uptake of 64Cu-ATSM in GB and its specificity for hypoxia, C6 glioma cells were selected based on our previous studies showing that this preclinical model presents a pronounced hypoxic component [10, 28].

**Fig. 1:**
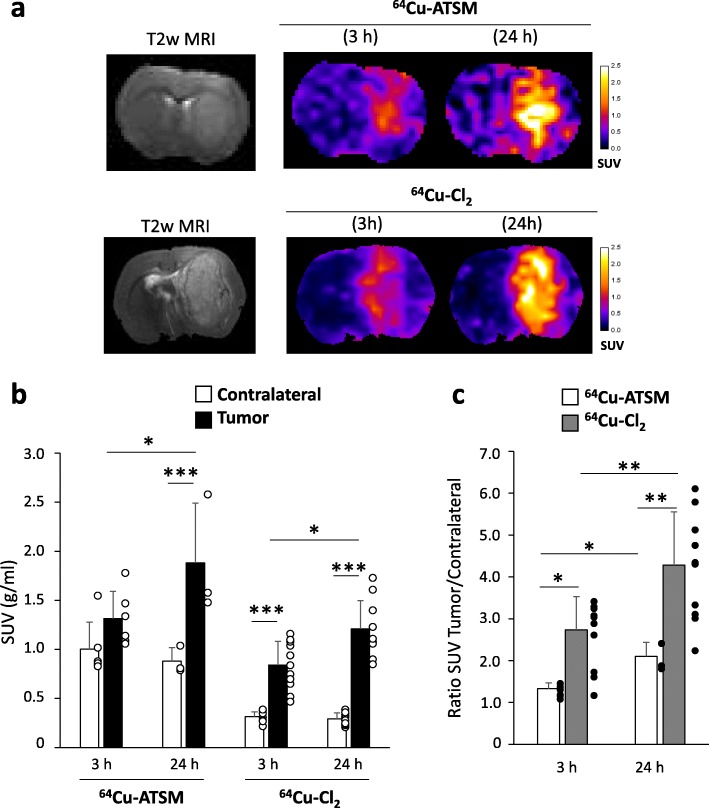
In vivo uptake of ^64^Cu-ATSM and ^64^Cu-Cl_2_ in C6 glioblastoma model. **a** MRI and PET images were acquired 15 days after tumor cell implantation into the striatum. ^64^Cu-ATSM and ^64^Cu-Cl_2_ uptake were evaluated at early (3 h) and late (24 h) times after radiotracer injection. Quantification of ^64^Cu-ATSM and ^64^Cu-Cl_2_ uptake from ipsilateral (tumor) and contralateral (healthy brain) SUVs (**b**) or ratio between tumor and contralateral SUVs (**c**) at the different imaging points. Mean ± SD, *n* = 6 rats for ^64^Cu-ATSM and *n* = 11 rats for ^64^Cu-Cl_2_. Tukey’s HSD test after significant two-ways ANOVA (group and time factors): *p < 0.05, **p < 0.01, and ***p < 0.001

For both ^64^Cu-ATSM and ^64^Cu-Cl_2_, the initial uptake was detectable as early as 3 h following injection and, intriguingly, kept increasing until 24 h (Fig. 1a, b). Quantitatively, for ^64^Cu-ATSM, in the healthy brain, SUV was 1.00 ± 0.28 g/mL at 3 h and 0.88 ± 0.14 g/mL at 24 h post-injection. In the tumor, SUV was greater relative to the healthy brain reaching 1.31 ± 0.28 g/mL at 3 h (non-significant, *p* = 0.33 compared to contralateral) and 1.88 ± 0.61 g/mL at 24 h (*p* < 0.0001 relative to contralateral) (Fig. 1b). ^64^Cu-ATSM SUV quantified at 24 h in tumor was significantly higher compared to 3 h post-injection (*p* < 0.05) whereas contralateral SUV did not change over time (Fig. 1b). The increase in SUV over time is also displayed in the time activity curve (TAC) (Additional file 1: Figure S2A). Blood samples collected over a period of 2 h demonstrated a biphasic curve. After the initial peak observed 12 s after the injection and a very rapid decay to reach a minimum at 7 min 30 s, values tended to increase thereafter until the end of measurements (i.e., 2 h) (Additional file 1: Figure S2B). Interestingly, the scatterplot of blood activity and values in the tumor obtained during the first 2 h were correlated (*R*^2^ = 0.93) (Additional file 1: Figure S2B).

Concerning ^64^Cu-Cl_2_, in the normal healthy brain, SUV was 0.31 ± 0.05 g/mL and 0.29 ± 0.06 g/mL at 3 h and 24 h respectively. These values were significantly lower than those observed for ^64^Cu-ASTM at 3 h and 24 h (Fig. 1b). In the tumor, ^64^Cu-Cl_2_ SUV was 0.84 ± 0.24 g/ml at 3 h (*p* < 0.001) and 1.21 ± 0.29 g/mL at 24 h (*p* < 0.001 relative to contralateral). As for ^64^Cu-ATSM, ^64^Cu-Cl_2_SUV significantly increased over time in the tumor (*p* < 0.05). The uptake in the tumor was significantly greater for ^64^Cu-ATSM than for ^64^Cu-Cl_2_ at 3 h and 24 h (Fig. 1b). Of note, as a result of the low uptake observed in the healthy tissue, the tumor/contralateral ratio was greater for ^64^Cu-Cl_2_ than ^64^Cu-ATSM at both 3 h (*p* < 0.05) and 24 h (*p* < 0.01) (Fig. 1c).

### Retention of Cu-ATSM and Cu-Cl_2_ and hypoxia

#### In vitro results

**Fig. 2:**
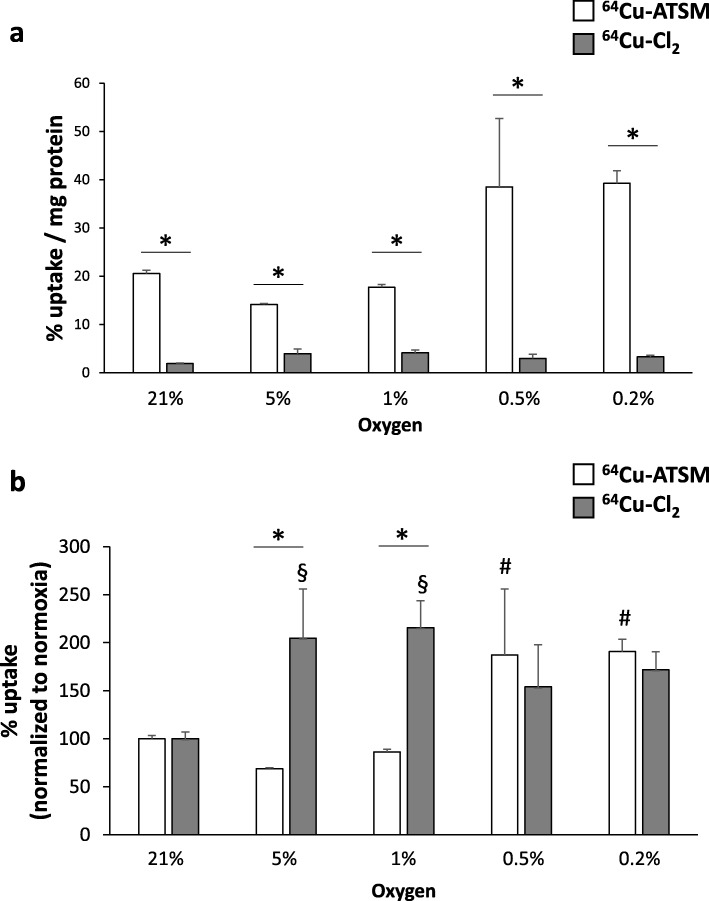
In vitro uptake of ^64^Cu-ATSM and ^64^Cu-Cl_2_ in tumor cells as a function of the degree of oxygenation. Quantification of cell retention of ^64^Cu-ATSM or ^64^Cu-Cl_2_ in normoxic (21% O_2_), moderate hypoxia (5% and 21% O_2_), or severe hypoxia (0.5% and 0.2% O_2_) for raw values (**a**) or normalized values relative to normoxic condition (**b**). Mean ± SD, *n* = 3 different cell cultures per condition. Tukey’s HSD test after significant two-ways ANOVA (group and time factors) was used. For comparison between ^64^Cu-ATSM and ^64^Cu-Cl_2_ for each oxygen condition, statistical significance was *p < 0.05. Statistical significances relative to normoxia were § < 0.05 for ^64^Cu-ATSM and ^#^*p* < 0.05 for ^64^Cu-Cl_2_

The relationship between 64Cu-ASTM and ^64^Cu-Cl_2_ uptake and hypoxia was analyzed in vitro at various oxygen levels (from 0.1 to 21% of oxygen). As expected, the uptake of ^64^Cu-ATSM was significantly greater than that of ^64^Cu-Cl_2_ both in normoxic and in hypoxic conditions (Fig. 2a). As instance, at 21% of oxygen, ^64^Cu-ATSM retention in C6 cells was 20.6 ± 0.7% uptake/mg protein whereas for ^64^Cu-Cl_2_, it was 1.9 ± 0.1% uptake/mg protein (*p* < 0.01). To better visualize the effect of hypoxia on cell retention, raw data were normalized relatively to the values obtained in normoxia for these two radiotracers (Fig. 2b). Interestingly, ^64^Cu-ATSM showed an increase in the uptake only in severe hypoxia at 0.5 and 0.2% of oxygen (187 ± 69% and 191 ± 13% respectively, compared to normoxia, *p* < 0.05) (Fig. 2b). Concerning ^64^Cu-Cl_2_, the cell retention was significantly increased at 5% and 1% of oxygen with no significant rise at lower oxygen percentages (Fig. 2b). Otherwise, the cellular uptake was not dependent on the incubation time (1 h or 4 h) for both radiotracers as mentioned in Additional file 1: Figures S3A and S3B. The hypoxia preconditioning time (3 h or 24 h before treatment with the radiotracers) had no effect (data not shown).

#### In vivo results

**Fig. 3:**
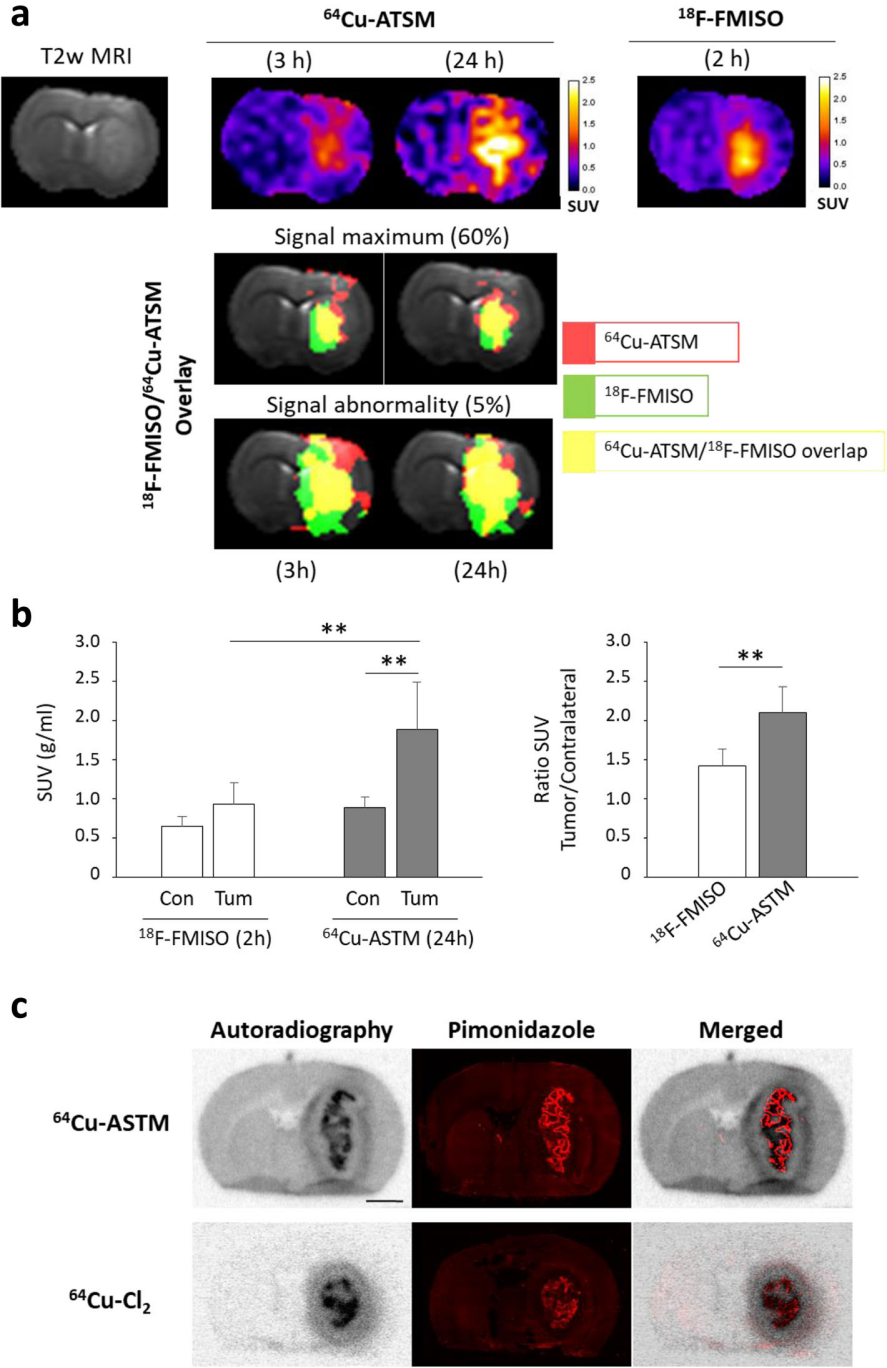
Relationships between retention of ^64^Cu-ATSM and ^64^Cu-Cl_2_ and hypoxia. **a** Co-registration of ^18^F-FMISO PET signal (2 h post-injection) with ^64^Cu-ATSM PET signal (3 h or 24 h post-injection). The overlaid images corresponds to 60% signal maximum or 5% signal abnormality for ^64^Cu-ATSM (red), ^18^F-FMISO (green), or ^64^Cu-ATSM/^18^F-FMISO merged (yellow). **b** ^18^F-FMISO and ^64^Cu-ATSM uptake were evaluated at 2 h and 24 h after radiotracer injection, respectively. Quantification of ^64^Cu-ATSM and ^18^F-FMISO uptake from ipsilateral (tumor) and contralateral (healthy brain) SUVs (left part) or ratio between tumor and contralateral SUVs (right part) at the different imaging points. Mean ± SD, *n* = 5 rats for ^18^F-FMISO and *n* = 3 rats for ^64^Cu-ATSM. Tukey’s HSD test after significant two-ways ANOVA (radiotracer and ROI factors): **p < 0.01. **c** From same coronal rat brain sections performed 24 h after ^64^Cu-ATSM or ^64^Cu-Cl_2_ injection, autoradiography and pimonidazole immunostaining were done to evaluate overlapping of these two labeling. Scale bar = 250 μm

^64^Cu-ATSM uptake was compared in the C6 GB model to ^18^F-FMISO. PET images obtained 2 h after ^18^F-FMISO injection were compared to ^64^Cu-ATSM signals acquired at an early (3 h) or a late (24 h) post-injection time and were overlaid on the T2w IRM images used to delimit tumor masses (Fig. 3a). Interestingly, a large spatial overlap was observed between ^18^F-FMISO and ^64^Cu-ATSM by comparing 60% maximum signal or 5% signal abnormality. At this late time point, an elevated uptake of the tracer was observed with SUV values larger than those of ^18^F-FMISO (^18^F-FMISO SUV = 0.93 ± 0.28 g/mL and ^64^Cu-ATSM SUV = 1.88 ± 0.61 g/mL, *p* < 0.05). As showed on Fig. 3b, a similar uptake of ^18^F-FMISO and ^64^Cu-ATSM in healthy brain was observed (SUV = 0.64 ± 0.12 g/ml and 0.88 ± 0.14 g/ml, respectively). In the tumor, SUV for ^18^F-FMISO was slightly increased relative to the healthy brain reaching 0.93 ± 0.28 g/ml (non-significant, *p* = 0.17) whereas SUV for ^64^Cu-ATSM was significantly higher than the contralateral brain (SUV tumor = 1.88 ± 0.61 g/ml compared to healthy tissue, *p* < 0.01). Ultimately, ^64^Cu-ATSM uptake was greater than ^18^F-FMISO in tumor (*p* < 0.01) as evidenced by the quantification of ratio tumor/contralateral: ratio T/C = 1.42 ± 0.22 and 2.10 ± 0.33 for ^18^F-FMISO and ^64^Cu-ATSM, respectively (*p* < 0.01) (Fig. 3b).

As presented in Fig. 3c, autoradiography confirmed a ^64^Cu-ATSM hypoxia-dependent tumor uptake as shown by spatial overlapping between autoradiography and pimonidazole staining. A similar result was observed for ^64^Cu-Cl_2_. The large difference in activity uptake in healthy brain between ^64^Cu-ATSM and ^64^Cu-Cl_2_ already observed in PET images was confirmed by autoradiography (Fig. 3c) and may be explained by the more lipophilic nature of ^64^Cu-ATSM compared to ^64^Cu-Cl_2_.

**Fig. 4:**
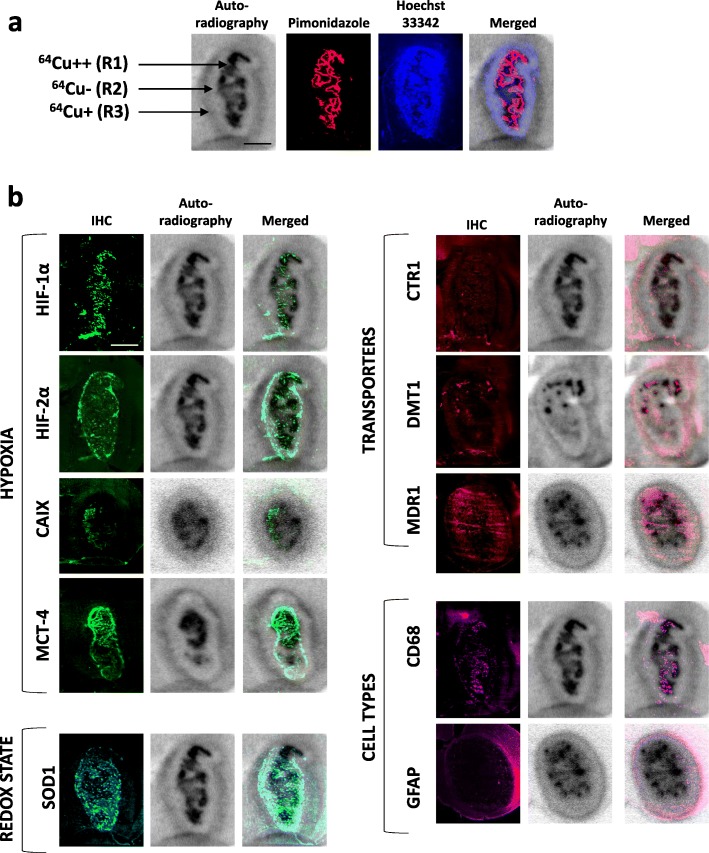
Relationships between the spatial heterogeneity of ^64^Cu uptake and hypoxia and expression of copper transporters. **a** Three areas with different intensities for ^64^Cu were distinguishable on the autoradiography: highly labeled area R1 (^64^Cu++), an unstaining intermediate region R2 (^64^Cu− and in the tumor periphery a moderate labeled region (^64^Cu+). Autoradiography was co-registered with pimonidazole staining to evaluate severe hypoxia and also Hoechst 33342 nucleic acid stain to identify the tumor mass. Scale bar = 200 μm. **b** Representative immunostaining of hypoxia markers (HIF-1α, HIF-2α, CAIX, MCT-4), specific or non-specific copper transporters (CTR1, DMT1, and MDR1), redox state (SOD1), and different cell types (CD68 for monocytes/macrophages and GFAP for reactive astrocytes). The autoradiography and immunohistochemistry studies were performed on same animals and consecutive sections. Scale bar = 200 μm

Interestingly, when looking at ^64^Cu-ATSM or ^64^Cu-Cl_2_ autoradiograms 24 h after the radiotracer injection, a spatial heterogeneity was observed and three distinct regions could be delimitated: (i) a central, highly labeled area corresponding to the dense tumor core and hypoxic tumor as observed on the Hoechst 33342 and pimonidazole staining (thereafter termed R1, ^64^Cu++); (ii) an intermediate region with no ^64^Cu labeling corresponding to a zone with high tumor cell density as seen on the Hoechst 33342 staining but without severe hypoxia as attested by pimonidazole staining (termed R2, ^64^Cu−); and (iii) a moderately labeled region at the periphery of the tumor which is not hypoxic, termed thereafter R3, ^64^Cu+ (Fig. 4a). To summarize, three areas could be delimitated by autoradiography: R1 (^64^Cu++/PIMO ++/Hoechst ++), R2 (^64^Cu−/PIMO−/Hoechst++), and R3 (^64^Cu+/PIMO−/Hoechst+). A similar regionalization of signal intensity for ^64^Cu was observed in the 3-h autoradiography (Additional file 1: Figure S4).

The ^64^Cu positive or negative regions were further characterized by immunohistochemistry in relation to the expression of several hypoxia and/or copper-related proteins in both ^64^Cu-ATSM or ^64^Cu-Cl_2_ injected animals (Fig. 4b). First, we compared the ^64^Cu uptake to hypoxic markers, namely HIF-1α, HIF-2α, CAIX, and MCT-4, by immunostaining (Fig. 4b). As shown in Fig. 4b, R1 (^64^Cu++) nicely superimposed to HIF-1α, CAIX, and MCT-4 staining. HIF-2α staining was observed in R1 (^64^Cu++) and mainly in R2 (^64^Cu−). Then, copper transporters were tested: the specific membrane transporter CTR1 (copper transporter 1) and the non-specific membrane transporters such as DMT1 (divalent metal transporter) and MDR1 (multidrug resistance protein 1). DMT1 was strongly expressed in R1 (^64^Cu++) and R3 (^64^Cu+) (Fig. 4b). CTR-1 was expressed mainly in R3 (^64^Cu+) while MDR1 was expressed in R1 (^64^Cu++) and R2 (^64^Cu−) (Fig. 4b). In parallel, SOD1 (superoxide dismutase 1 Cu-Zn) expression was studied because previous studies in other cancer types had suggested that redox state of tumor cells could be involved in ^64^Cu-ATSM uptake. In this study on GB, SOD1 was mainly present in area R2 (^64^Cu−) (Fig. 4b). Lastly, we analyzed some cellular reactions known to occur in parallel to tumor growth namely astrogliosis and inflammation and which might be related to ^64^Cu-ATSM or ^64^Cu-Cl_2_ uptake. CD68 and GFAP (glial fibrillary acidic protein) staining were used to assess inflammation and astrogliosis, respectively. The CD68 staining was higher in R1 (^64^Cu++) whereas GFAP staining was greater in R3 (^64^Cu+) (Fig. 4b).

**Fig. 5:**
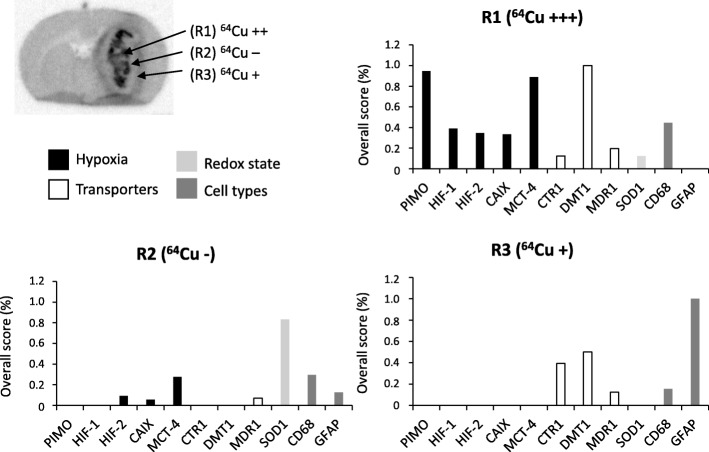
Quantification of immunostaining for hypoxia, transporters, redox state, and cell types according to the spatial distribution of ^64^Cu uptake. For each region defined on the autoradiography (R1 (^64^Cu++), R2 (^64^Cu−), and R3 (^64^Cu+)), an overall score was measured by multiplying the number of positive samples and the intensity score for each immunolabeling (*n* = 9 rats)

For each region, the immunostaining was quantified according to two different parameters: the number of positive samples and the intensity score by attributing 1 point for low signal or 2 points for high signal (Additional file 2: Table S3). To have an overview of the different labeling, an overall score was measured by multiplying the number of positive samples and the intensity score (Fig. 5). Briefly, the overall score highlighted that the high intensity of ^64^Cu (R1) was strongly correlated to the uptake of pimonidazole and the expression of MCT-4 and DMT1; the moderate intensity of ^64^Cu (R3) corresponds to astrogliosis that also expressed CTR1 and DMT1.

### Expression of copper transporters (influx) as a function of hypoxia

**Fig. 6:**
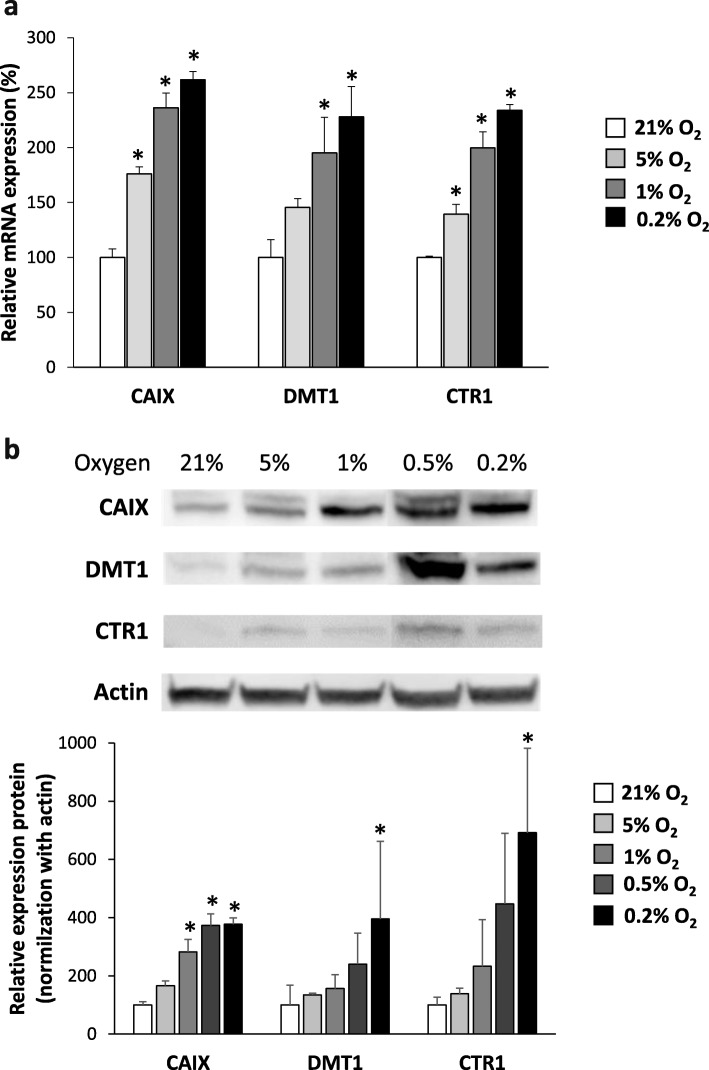
Expression of copper transporters, CTR1 and DMT1, in hypoxia. Quantification of expression of CTR1 and DMT1 at mRNA (**a**) or protein levels (**b**) from cells exposed for 24 h in hypoxic conditions (5%, 1%, 0.5%, and 0.2% O_2_) relative to normoxia (21% O_2_). CAIX expression was used as positive hypoxic control. Mean ± SD, *n* = 3 different cell cultures per condition. Tukey’s HSD test after significant one-way ANOVA: *p < 0.05

To further address the dependency of copper transporters on hypoxia, we assessed the expression of both DMT1 and CTR1 at the mRNA (Fig. 6a) and protein levels (Fig. 6b). CAIX expression was used as a positive hypoxic control.

At the mRNA level, CAIX expression was significantly increased at 5% of oxygen and kept increasing at lower oxygen percentages (Fig. 6a). DMT1 was significantly overexpressed at 1% and 0.2% of O_2_ while CTR1 was overexpressed at 5% of oxygen and kept increasing at oxygen percentage. Similar trends were observed at the protein level for the three proteins studied (Fig. 6b). Interestingly, we demonstrated that copper transporter expression is strongly dependent of oxygen level (Additional file 1: Figure S5). The protein expression of DMT1 and CTR1 are almost return at the baseline as soon as 6 h of reoxygenation. The results sustain that if a reperfusion occurs in case of transient hypoxia in the tumor, copper transporter expression rapidly return to basal level.

## Discussion

Cancer patients ought to benefit from hypoxia mapping to deal with hypoxia-induced radioresistance and chemoresistance. ^64^Cu-ATSM is a PET radiopharmaceutical that exhibits a low redox potential. It has been proposed as a selective marker of tissue hypoxia. In the present study, using a combination of both in vitro and in vivo experiments, we showed that ^64^Cu-ATSM accumulates in severe hypoxia (pimonidazole positive areas in vivo and less than 1% of oxygen in vitro). However, an uptake of ^64^Cu-ATSM was also observed at the outer periphery of the tumor where no evidence of hypoxia was detected (pimonidazole, HIF-1α, HIF-2α, CAIX, and MCT4 negative) but where specific cellular reactions such as the astrogliosis were observed. Interestingly, an increase in the expression of copper transporters was detected in this region. We also observed a marked uptake of ^64^Cu-Cl_2_ in tumors with a similar uptake regionalization. Interestingly, for both tracers, the uptake was more pronounced at 24 h than at 3 h post-injection. However, greater uptake values, measured in vivo and also in vitro, confirmed the interest of ^64^Cu-ATSM compared to ^64^Cu-Cl_2_. So, ^64^Cu-*ATSM* is actually a neutral *lipophilic* molecule with high cell membrane permeability that diffuses readily from the bloodstream to surrounding cells.

One of the first observations in this study was the uptake of both ^64^Cu-ATSM (and ^64^Cu-Cl_2_) in C6 glioma tumors. This uptake was detected in the tumor core, a region where nitroimidazole uptake was also detected. In previous studies, we already demonstrated a pronounced uptake of both ^18^F-FMISO and pimonidazole in this C6 glioma model [10, 28], and oxygen measurements with EPR probes confirmed that ptO_2_ was less than 10 mmHg of oxygen [29]. The present results highlight that ^64^Cu-ATSM (and ^64^Cu-Cl_2_) could be considered as selective tracers of hypoxia, which is in line with previous studies performed in the flank of rats that demonstrated correlations between ^64^Cu-ATSM and ^18^F-FMISO uptakes and between ^64^Cu-ATSM uptake and low pO2 [13, 14]. However, autoradiography also demonstrated that uptake occurred in regions where severe hypoxia was not evidenced (region R3). This observation raises some question about the specificity of ^64^Cu complexes as already discussed [30]. In our experiments, we observed similarities between ^64^Cu-ATSM and ^64^Cu-Cl_2_ uptake in the tumor hypoxic core (R1). These observations have already been described in head and neck cancer models and human colorectal models [31]. Similar behaviors have already been reported when comparing Cu-ATSM and Cu-Acetate [32]. As discussed, these data support the idea that the in vivo uptake may be the reflection of copper metabolism by the host organism and the tumor cells [22].

The expression of copper transporters may be increased by hypoxia and that may also explain the similar uptake of both ^64^Cu-ATSM and ^64^Cu-Cl_2_. In the ex vivo study, we confirmed that DMT1 is overexpressed in the pimonidazole positive region and that both DMT1 and CTR1 are also expressed in the outer ring of the tumor where pimonidazole staining is negative. These results support the idea that, while Cu-complexes could be retained in hypoxic cells following redox metabolism, they could also be detected in non-hypoxic cells expressing high amounts of transporters. Various situations may explain an increase in copper transporters. For instance, hypoxia is known to induce the expression of DMT1 through the activation of HIF-1α [33, 34] and the expression of CTR1 through HIF-2α [35]. In vitro, we also confirm that the expression of both influx transporters for copper is increased by hypoxia. Interestingly, we also observed a correlation between the uptake of Cu-complexes, CTR1 and DMT1 expression, and the astrogliosis at the outer periphery of the tumor. Astrocytes have already been described as a cornerstone in copper metabolism in the brain [36]. It is known that cultured astrocytes efficiently take up copper ions predominantly by the copper transporter CTR1 and the divalent metal transporter DMT1 [37]. In the study of Jorgensen and colleagues [38], the authors did not show correlation between the uptake of ^64^Cu and the expression of CTR1. However, when CTR1 was overexpressed in human breast cancer cells, it resulted in an increased uptake of ^64^Cu-Cl_2_ [39] while inhibition of CTR1 in prostate cancer cells resulted in a decreased uptake of ^64^Cu-Cl_2_ [40]. These data highlight the relationships that exist between copper transporters and imaging results. Interestingly, in the context of GB, hypoxia is well known as a poor prognosis factor. We analyzed the relationships between copper transporters and patient survival using the REMBRANDT database. We observed that CTR1 expression is also predictive of patient survival (Additional file 1: Figure S6A) but not DMT1 (Additional file 1: Figure S6B). No study has so far analyzed the dependency of DMT1 expression on Cu-complex uptake.

Another observation was the increase in ^64^Cu-ATSM and ^64^Cu-Cl_2_ uptake between 3 h and 24 h post-injection. This observation was in line with the arterial concentrations of ^64^Cu. While the initial exponential decay following the peak that occurred at about 7 min, an increase occurred thereafter and the blood activity kept increasing until the end of the measurements. One explanation lies on the fact that organs like liver could metabolize the ^64^Cu-ATSM complex and non-tracer-bound radioactive copper that may influence PET measurements could be transported into the blood stream, stored in hepatobiliary stores and released thereafter [38, 41] as either free ^64^Cu or protein-bound (albumin, ceruloplasmin… )[42, 43]. This metabolic pathway has been confirmed using D-penicillin which reduced liver uptake of Cu-ATSM [44]. Therefore, late PET imaging is influenced by the metabolic pathway.

Lastly, the development of novel therapeutic approaches to GB is essential and those based on radionuclide therapy could be of significant clinical impact. It was proposed in preclinical models that ^64^Cu-Cl_2_ [45] and also ^64^Cu-ATSM [46] could be pertinent theranostic agents for brain tumors. In this direction, the present study suggests a double interest in using ^64^Cu complexes internal radiotherapy for GB: target hypoxic areas (region R1 in the autoradiography) as well as the invasive areas localized at the periphery of tumors that are very rich in reactive astrocytes because of the invasion of tumor cells (region R3 on the autoradiography).

Considering hypoxia and based on our results, Cu-ATSM offers some benefits to FMISO. As an example, tumor to background ratio is greater owing to its lipophilicity. Cu-ATSM may enable to see hypoxia in brain tumors with intact BBB. Thanks to its elevated half-life, ^64^Cu offers widespread dissemination.

Our results show that the uptake is mediated through the overexpression of copper transporters induced by changes in oxygen tension.

## Conclusions

As a conclusion, in the present study, we show that Cu complexes undoubtedly accumulate in hypoxic areas of the tumors. This uptake may be the reflection of a direct dependency to a redox metabolism and also a reflection of hypoxic-induced overexpression of transporters. We also show that Cu-ATSM also stained, to a lesser extent, non-hypoxic regions with high expression of copper transporters and in particular DMT-1 and CTR1 such as astrogliosis.

## Supplementary information

**Additional file 1: Figure S1.** Schematic representation of the protocol used for in vivo experiments. **Figure S2.** Evaluation of ^64^Cu-ATSM activity in the brain and the plasma. **(A)** Time activity curve (TAC) measured for 24 hours after the radiotracer injection in the tumor, peritumoral area and healthy brain. **(B)** Superposition of TAC in the blood and in the tumor quantified during 2 hours after ^64^Cu-ATSM injection. **Figure S3.** In vitro uptake of ^64^Cu-ATSM and ^64^Cu-Cl_2_ in tumor cells according the time incubation with radiotracer. Quantification of cell retention of ^64^Cu-ATSM **(A)** and ^64^Cu-Cl_2_ **(B)** in normoxic (21% O_2_) or hypoxic (0.5% and 0.2% O_2_) conditions after 1 hour or 4 hours of incubation of the tumor cells with the radiotracer brought into the culture medium. Mean ± SD, *n* = 3 different cell cultures per condition. Tukey’s HSD test after significant two-ways ANOVA (oxygen and time factors) was used: no significant difference was obtained. **Figure S4.** Spatial distribution of ^64^Cu-ATSM uptake in autoradiography at 3 hours or 24 hours after radiotracer injection. **Figure S5.** Protein expression of copper transporters, CTR1 and DMT1, in transient hypoxia. Cells were exposed to hypoxia (0.2% O_2_) during 24 hours and then reoxygenated (21%O_2_) during different times (6, 24 or 24 hours). Representative western-blot of DMT1 and CTR1 (A) and quantification of their protein expression (B). CAIX expression was used as positive hypoxic control. Mean ± SD, *n* = 3 different cell cultures per condition. Tukey’s HSD test after significant one-way ANOVA: * *p* < 0.05. **Figure S6.** Survival analyses of patients with glioblastoma according to the expression of copper transporters CTR1 and DMT1. Kaplan-Meier survival plot of glioblastoma patients were assessed according to the level of CTR1 or DMT1 gene expression from the REMBRANT database by using Betastasis online software (http://www.betastasis.com/, date of last access: 4.2.2012).

**Additional file 2: Table S1.** Details of primary and secondary antibodies used. IF=immunofluorescence, WB=western-blot. **Table S2.** Details of rat primers used for RT-qPCR analysis. **Table S3.** Quantification of immunostaining performed on brain slices with ^64^Cu-ATSM or ^64^Cu-Cl_2_ uptake in different areas R1, R2 and R3. Numbers in bold reflect immunolabeling greater than 75%.

## Data Availability

The datasets used and/or analyzed during the current study are available from the corresponding author on reasonable request.

## Abbreviations

^18^F-FMISO: 3-^18^F-fluoro-1-(2-nitro-1-imidazolyl)-2-propanol
^64^Cu-ATSM: Diacetyl-bis(N4-methylthiosemicarbazone)
CAIX: Carbonic anhydrase IX
CTR1: Copper transporter 1
DMT-1: Divalent metal transporter 1
HIFs: Hypoxia-inducible factors
MDR1: Multidrug resistance protein 1
