# Supplementary figures and images for: 64Cu-ATSM/64Cu-Cl2 and their relationship to hypoxia in glioblastoma: a preclinical study
Source: EJNMMI Res. 2019 Dec 19;9:114. doi: 10.1186/s13550-019-0586-6 (PMC6923301; doi:10.1186/s13550-019-0586-6)

FIGURE S1

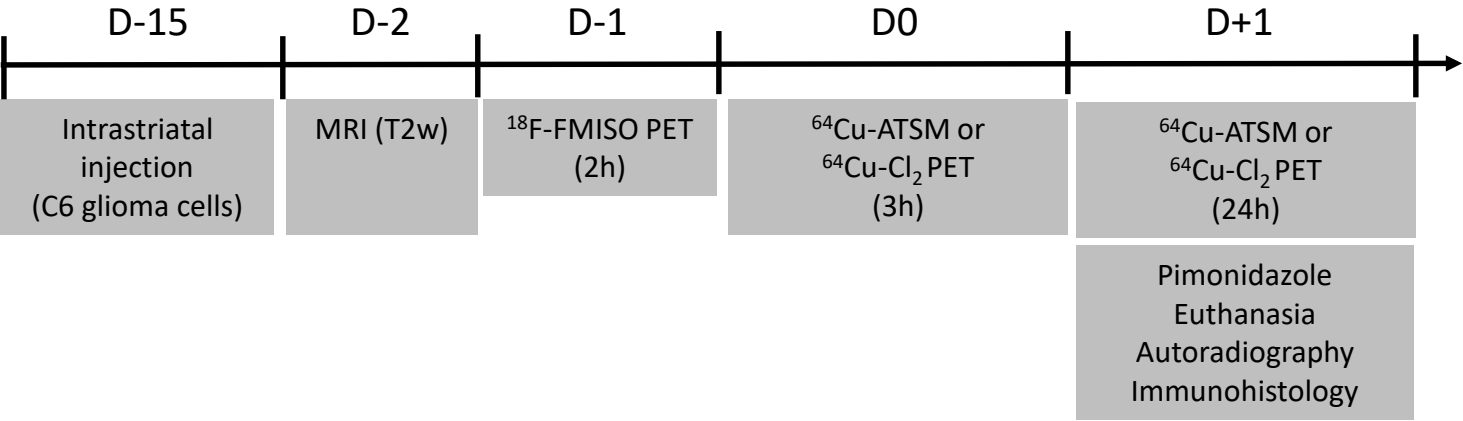

FIGURE S2

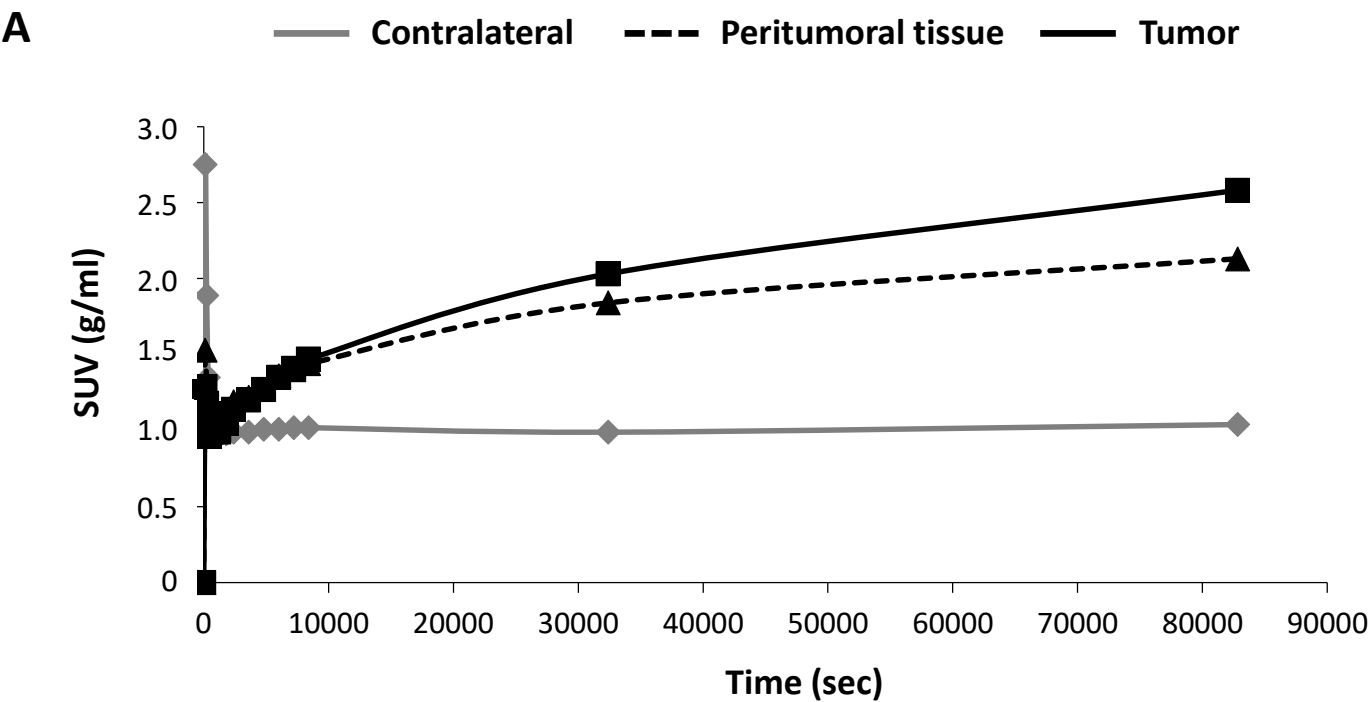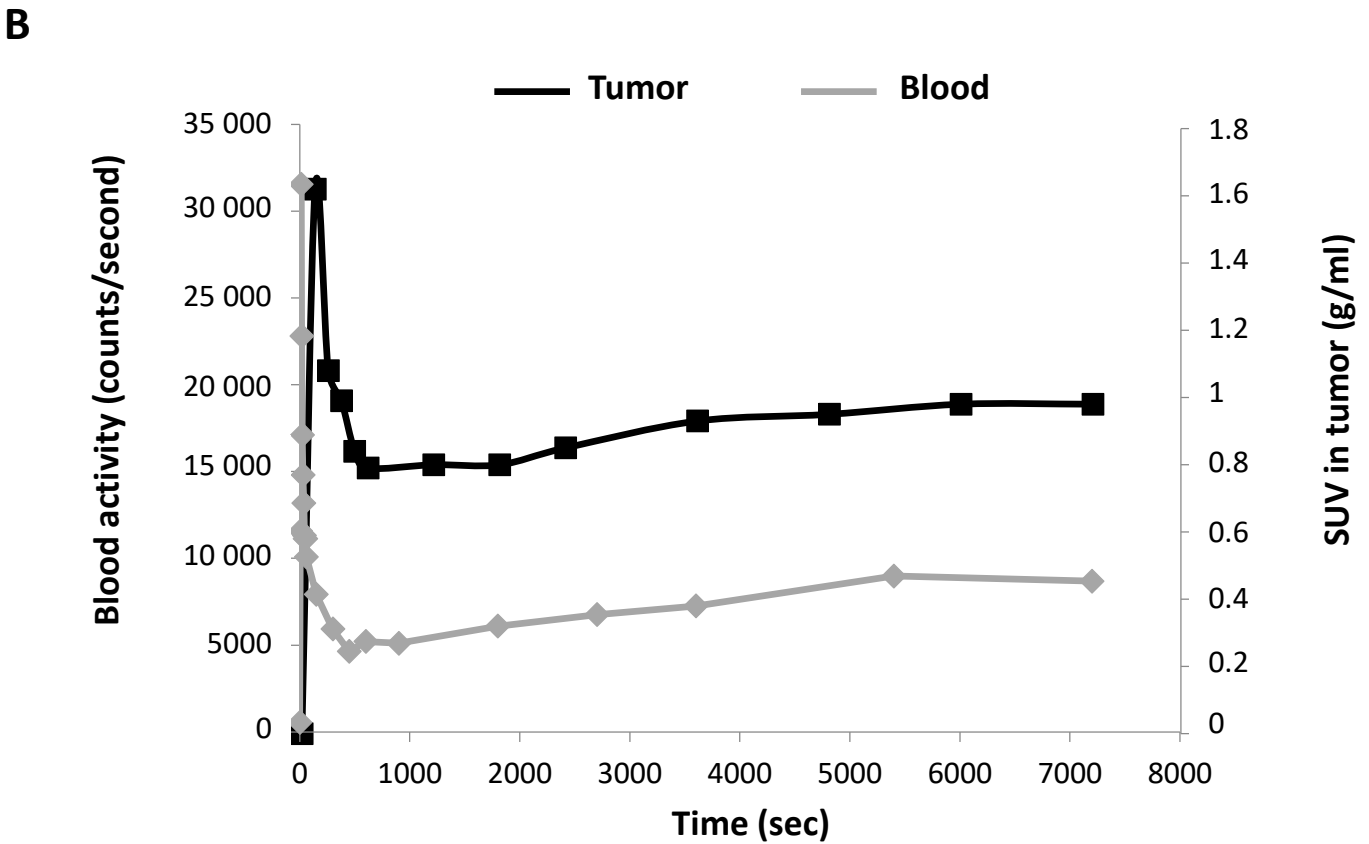

FIGURE S3

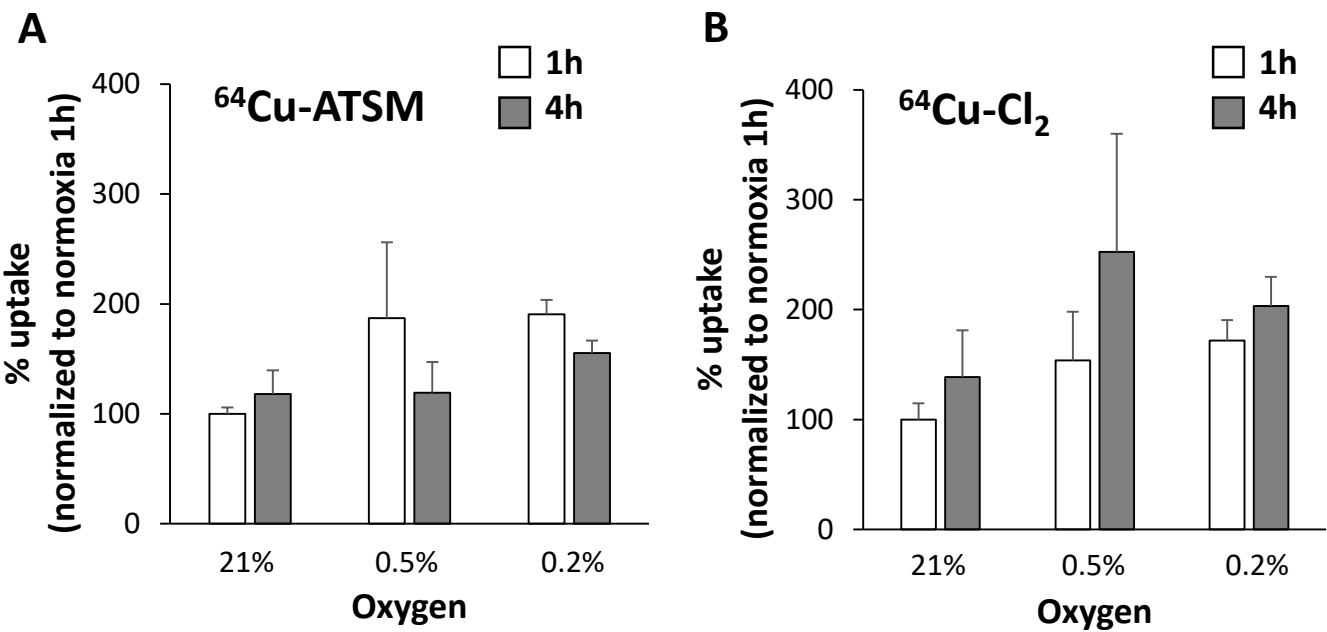

**FIGURE S4**

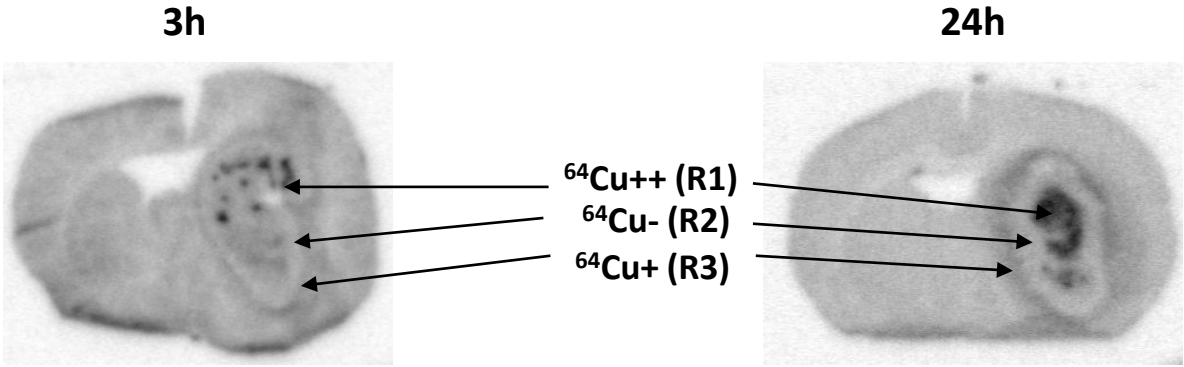

FIGURE S5

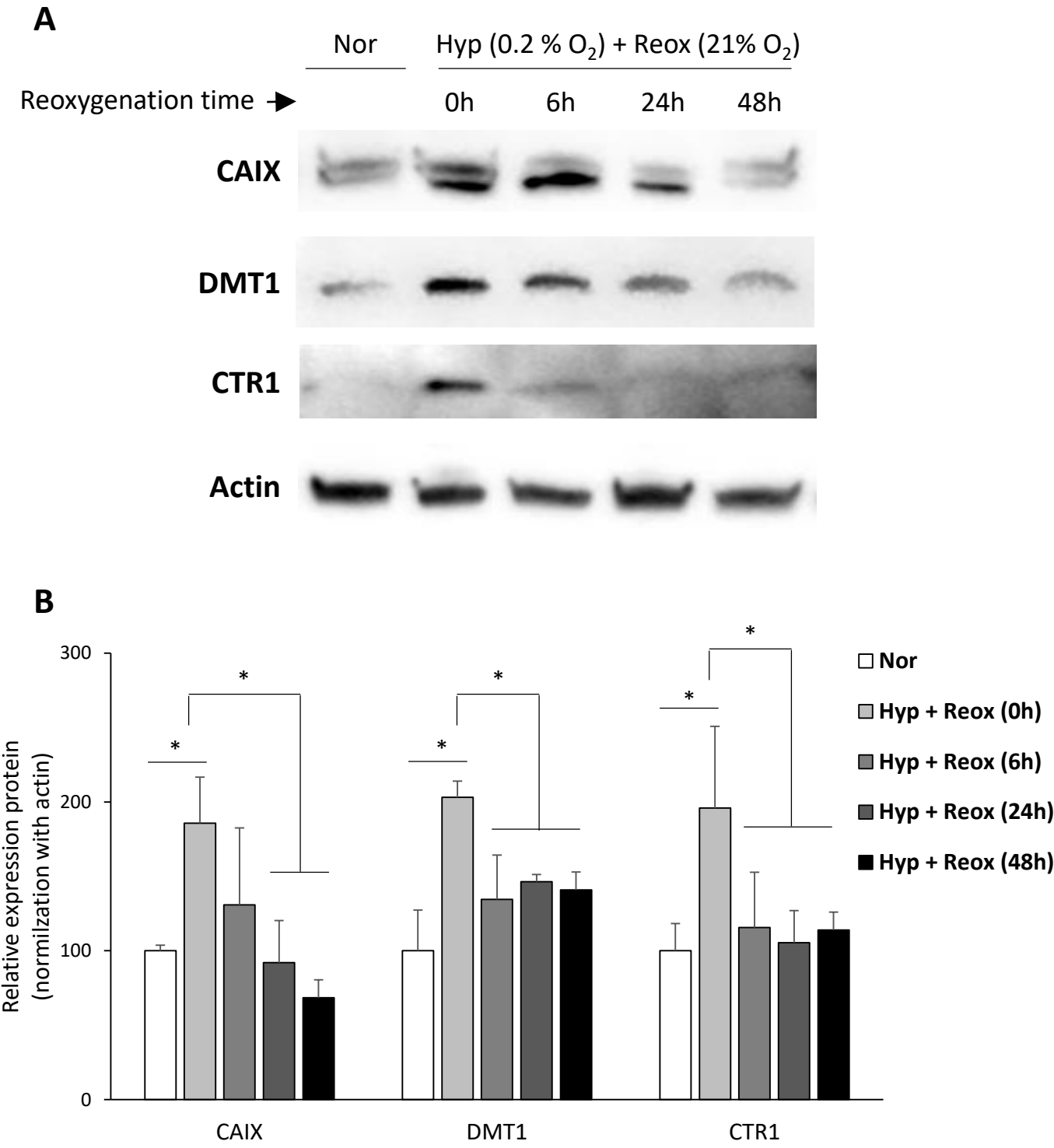

FIGURE S6

A CTR1 in glioblastoma

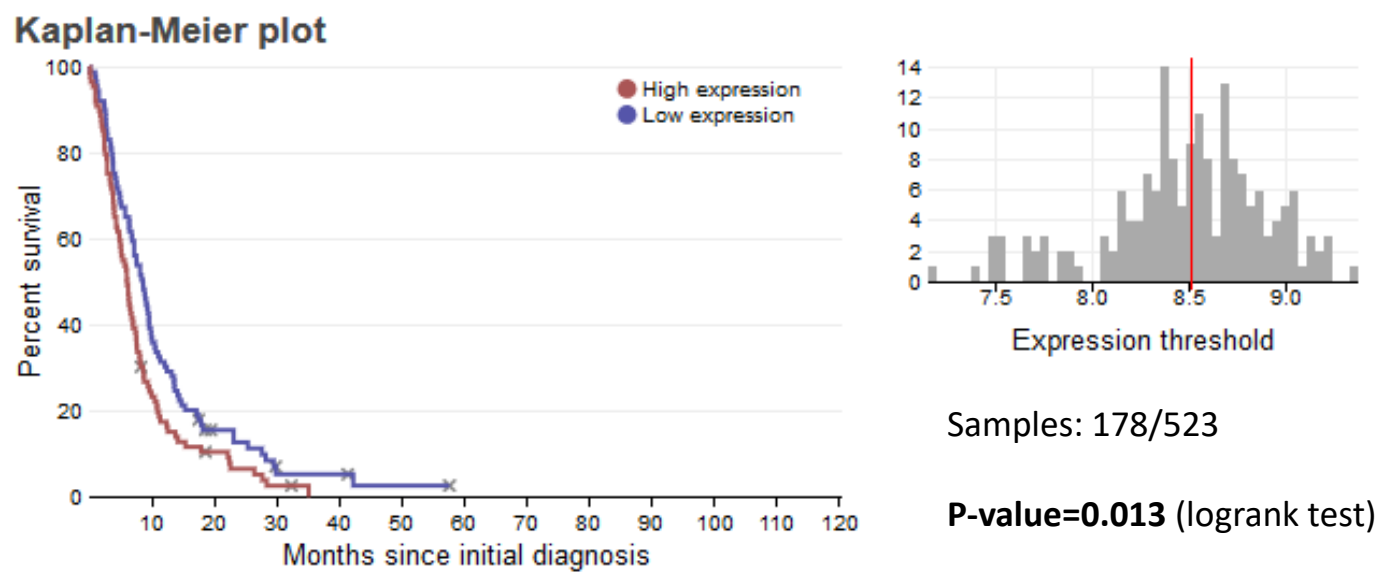

B DMT1 in glioblastoma

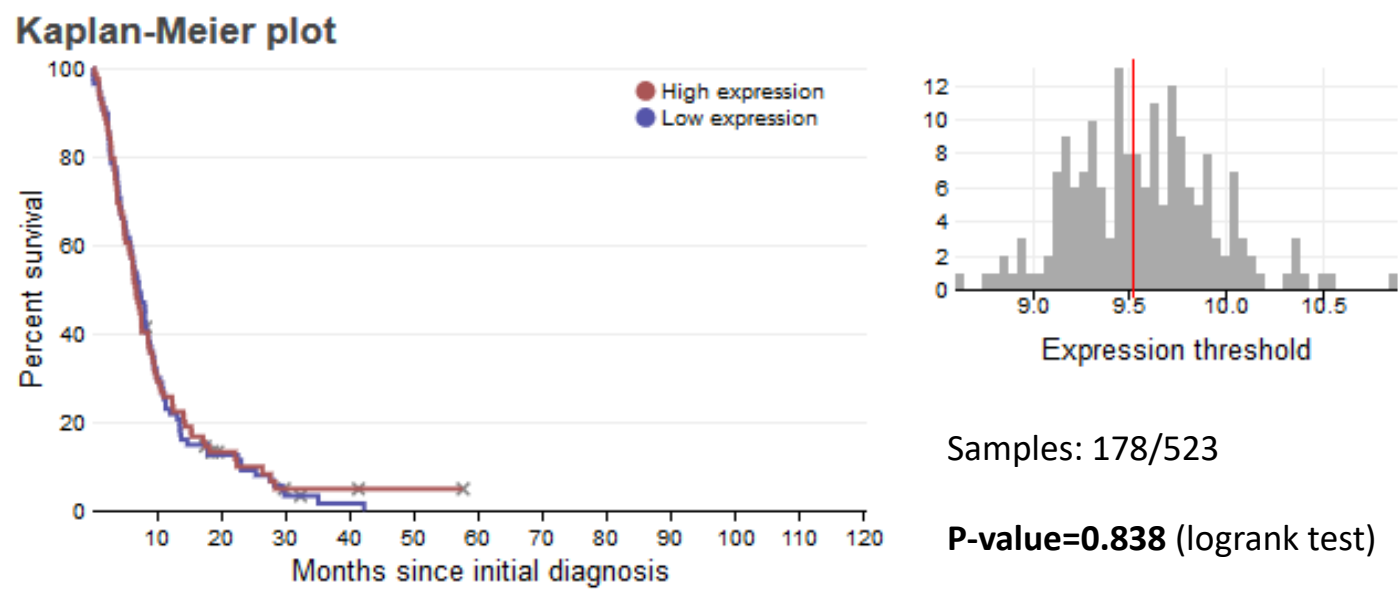

Supplement: Supplementary file 1 — Additional file 1: Figure S1. Schematic representation of the protocol used for in vivo experiments. Figure S2. Evaluation of 64Cu-ATSM activity in the brain and the plasma. (A) Time activity curve (TAC) measured for 24 hours after the radiotracer injection in the tumor, peritumoral area and healthy brain. (B) Superposition of TAC in the blood and in the tumor quantified during 2 hours after 64Cu-ATSM injection. Figure S3. In vitro uptake of 64Cu-ATSM and 64Cu-Cl2 in tumor cells according the time incubation with radiotracer. Quantification of cell retention of 64Cu-ATSM (A) and 64Cu-Cl2 (B) in normoxic (21% O2) or hypoxic (0.5% and 0.2% O2) conditions after 1 hour or 4 hours of incubation of the tumor cells with the radiotracer brought into the culture medium. Mean ± SD, n = 3 different cell cultures per condition. Tukey’s HSD test after significant two-ways ANOVA (oxygen and time factors) was used: no significant difference was obtained. Figure S4. Spatial distribution of 64Cu-ATSM uptake in autoradiography at 3 hours or 24 hours after radiotracer injection. Figure S5. Protein expression of copper transporters, CTR1 and DMT1, in transient hypoxia. Cells were exposed to hypoxia (0.2% O2) during 24 hours and then reoxygenated (21%O2) during different times (6, 24 or 24 hours). Representative western-blot of DMT1 and CTR1 (A) and quantification of their protein expression (B). CAIX expression was used as positive hypoxic control. Mean ± SD, n = 3 different cell cultures per condition. Tukey’s HSD test after significant one-way ANOVA: * p < 0.05. Figure S6. Survival analyses of patients with glioblastoma according to the expression of copper transporters CTR1 and DMT1. Kaplan-Meier survival plot of glioblastoma patients were assessed according to the level of CTR1 or DMT1 gene expression from the REMBRANT database by using Betastasis online software (http://www.betastasis.com/, date of last access: 4.2.2012). [file 13550_2019_586_MOESM1_ESM.pdf]
