# Supplementary material for: 64Cu-ATSM/64Cu-Cl2 and their relationship to hypoxia in glioblastoma: a preclinical study
Source: EJNMMI Res. 2019 Dec 19;9:114. doi: 10.1186/s13550-019-0586-6 (PMC6923301; doi:10.1186/s13550-019-0586-6)
Supplement: Supplementary file 2 — Additional file 2: Table S1. Details of primary and secondary antibodies used. IF=immunofluorescence, WB=western-blot. Table S2. Details of rat primers used for RT-qPCR analysis. Table S3. Quantification of immunostaining performed on brain slices with 64Cu-ATSM or 64Cu-Cl2 uptake in different areas R1, R2 and R3. Numbers in bold reflect immunolabeling greater than 75%. [file 13550_2019_586_MOESM2_ESM.pdf]

| Target | Dilution (application) | Supplier | Reference |
|---|---|---|---|
| Pimonidazole | 1:50 (IF) | Hypoxyprobe Inc | Hypoxyprobe™-1 Kit |
| HIF-1α | 1:50 (IF) | Novus Biologicals | NB100-105 |
| HIF-2α | 1:200 (IF) | Genetex | GTX30114 |
| CAIX | 1:500 (IF) / 1:2000 (WB) | Novus Biologicals | NB100-417 |
| MCT-4 | 1:100 (IF) | Santa Cruz Biotechnologies | sc-376140 |
| SOD1 | 1:200 (IF) | Novus Biologicals | NBPA-90186 |
| CTR1 | 1:250 (IF) / 1:1000 (WB) | Novus Biologicals | NB100-402 |
| DMT1 | 1:1000 (IF) / 1:200 (WB) | Abcam | ab55735 |
| MDR1 | 1:100 (IF) | Novus Biologicals | NB600-1036 |
| CD68 | 1:100 (IF) | Millipore | MAB1435 |
| GFAP | 1:500 (IF) | DAKO | 20334 |
| β-Actin | 1:1000 (WB) | Cell signaling | 3700 |
| Alexa Fluor® anti-rabbit 488 | 1:200 (IF) | Invitrogen | A-21206 |
| Alexa Fluor® anti-mouse 555 | 1:200 (IF) | Invitrogen | A-31570 |
| Anti-rabbit IgG peroxidase | 1:10000 (WB) | Sigma-Aldrich | A0545 |
| Anti-mouse IgG peroxidase | 1:5000 (WB) | Sigma-Aldrich | A4416 |

**Table S1: Details of primary and secondary antibodies used. IF=immunofluorescence, WB=western-blot**

| mRNA | Primer | Sequence |
|---|---|---|
| CAIX | Forward<br>Reverse | 5’-TCA CCT CAG TAC AGC CTT CTC A-3’<br>5’-GGC ACT GTT TTC TTC TGG ACT C-3’ |
| CTR1 | Forward<br>Reverse | 5’-TCG GCC TCA CAC TCC CAC GA-3’<br>5’-CGA AGC AGA CCC TCT CGG GC-3’ |
| DMT1 | Forward<br>Reverse | 5’-TCG CAG GCG GCA TCT TGG TC-3’<br>5’-TAC CGA GCG CCC ACA GTC CA-3’ |
| β-Actin | Forward<br>Reverse | 5’-GAC AGG ATG CAG AAG GAG ATT ACT-3’<br>5’-TGA TCC ACA TCT GCT GGA AGG T-3’ |

**Table S2: Details of rat primers used for RT-qPCR analysis.**

| | | R1 ($^{64Cu}$ ++) Positive samples | R1 Intensity score | R2 ($^{64Cu}$ -) Positive samples | R2 Intensity score | R3 ($^{64Cu}$ +) Positive samples | R3 Intensity score |
|---|---|---|---|---|---|---|---|
| Hypoxia | PIMO | **9/9 (100 %)** | **17/18 (94 %)** | 0/9 (0 %) | 0/18 (0 %) | 0/9 (0 %) | 0/18 (0 %) |
| | HIF-1 | **7/9 (78 %)** | 9/18 (50 %) | 1/9 (11 %) | 1/18 (6 %) | 0/9 (0 %) | 0/18 (0 %) |
| | HIF-2 | **7/9 (78 %)** | 8/18 (44 %) | 3/9 (33 %) | 5/18 (28 %) | 0/9 (0 %) | 0/18 (0 %) |
| | CAIX | 6/9 (67 %) | 9/18 (50 %) | 3/9 (33 %) | 3/18 (17 %) | 0/9 (0 %) | 0/18 (0 %) |
| | MCT-4 | **9/9 (100 %)** | **16/18 (89 %)** | 5/9 (56 %) | 9/18 (50 %) | 0/9 (0 %) | 0/18 (0 %) |
| Transporters | CTR1 | 4/9 (44 %) | 5/18 (28 %) | 1/9 (11%) | 1/18 (6%) | **8/9 (89%)** | 8/18 (44 %) |
| | DMT1 | **9/9 (100 %)** | **18/18 (100 %)** | 0/9 (0 %) | 0/18 (0 %) | **9/9 (100 %)** | 9/18 (50 %) |
| | MDR1 | 5/8 (63 %) | 5/16 (31 %) | 3/8 (38 %) | 3/16 (19 %) | 4/8 (50 %) | 4/16 (25 %) |
| Redox state | SOD1 | 4/9 (44 %) | 5/18 (28 %) | **9/9 (100 %)** | **15/18 (83 %)** | 0/9 (0 %) | 0/18 (0 %) |
| Macrophages | CD68 | 6/9 (67 %) | 12/18 (67 %) | 6/9 (67 %) | 8/18 (44 %) | 5/9 (56 %) | 5/18 (28 %) |
| Astrocytes | GFAP | 0/8 (0 %) | 0/16 (0 %) | 4/8 (50 %) | 4/16 (25 %) | **8/8 (100 %)** | **16/16 (100 %)** |

**Table S3: Quantification of immunostaining performed on brain slices with $^{64}Cu$-ATSM or $^{64}Cu$-$Cl_2$ uptake in different areas R1, R2 and R3.** Numbers in bold reflect immunolabeling greater than 75%.
